# Effectiveness of Interactive Digital Decision Aids in Prenatal Screening Decision-making: Systematic Review and Meta-analysis

**DOI:** 10.2196/37953

**Published:** 2023-03-14

**Authors:** Hong Yat Conrad Wong, Saba Asim, Qi Feng, Sherry Xiao-hong Fu, Daljit Singh Sahota, Po Lam So, Dong Dong

**Affiliations:** 1 The Jockey Club School of Public Health and Primary Care The Chinese University of Hong Kong Shatin Hong Kong; 2 Barts and The London School of Medicine and Dentistry Queen Mary University of London London United Kingdom; 3 Nuffield Department of Population Health University of Oxford Oxford United Kingdom; 4 Department of Obstetrics and Gynaecology The Chinese University of Hong Kong Shatin Hong Kong; 5 Department of Obstetrics and Gynaecology Tuen Mun Hospital Tuen Mun Hong Kong; 6 Shenzhen Research Institute The Chinese University of Hong Kong Shenzhen China

**Keywords:** informed decision-making, interactive digital decision aids, pregnancy, prenatal screening, systematic review

## Abstract

**Background:**

Increasing prenatal screening options and limited consultation time have made it difficult for pregnant women to participate in shared decision-making. Interactive digital decision aids (IDDAs) could integrate interactive technology into health care to a facilitate higher-quality decision-making process.

**Objective:**

The objective of this study was to assess the effectiveness of IDDAs on pregnant women’s decision-making regarding prenatal screening.

**Methods:**

We searched Cochrane Central Register of Controlled Trials, MEDLINE, Embase, PsycINFO, World Health Organization International Clinical Trials Registry Platform, Google Scholar, and reference lists of included studies until August 2021. We included the randomized controlled trials (RCTs) that compared the use of IDDAs (fulfilling basic criteria of International Patient Decision Aid Standards Collaboration and these were interactive and digital) as an adjunct to standard care with standard care alone and involved pregnant women themselves in prenatal screening decision-making. Data on primary outcomes, that is, knowledge and decisional conflict, and secondary outcomes were extracted, and meta-analyses were conducted based on standardized mean differences (SMDs). Subgroup analysis based on knowledge was performed. The Cochrane risk-of-bias tool was used for risk-of-bias assessment.

**Results:**

Eight RCTs were identified from 10,283 references, of which 7 were included in quantitative synthesis. Analyses showed that IDDAs increased knowledge (SMD 0.58, 95% CI 0.26-0.90) and decreased decisional conflict (SMD –0.15, 95% CI –0.25 to –0.05). Substantial heterogeneity in knowledge was identified, which could not be completely resolved through subgroup analysis.

**Conclusions:**

IDDAs can improve certain aspects of decision-making in prenatal screening among pregnant women, but the results require cautious interpretation.

## Introduction

Prenatal screening for common fetal chromosomal abnormalities such as trisomy 21, open neural tube defects, and specific inherited gene disorders is now routinely offered to all pregnant women [1]. It has been shown that the expansion of prenatal screening has managed to help lower the number of babies born with Down syndrome every year by an average of 54% in Europe, whereas in the United States, it is approximately 33% as a result of Down syndrome–related selective terminations [2].

The decision whether to undergo or decline the offer of prenatal screening and further prenatal diagnosis is multifaceted and dependent on their own and partners’ knowledge, values, social and familial acceptance, and willingness to care for any potentially affected offspring, and lastly, their personal and societal views in regard to termination of pregnancy [3]. Informed decision-making is now even more complex and challenging as screening and diagnosis pathway options have expanded to now be able to not only common aneuploidies but also much rarer aneuploidies using either cell-free DNA in maternal blood or DNA from chorionic villus samples or amniocytes.

Available studies would, however, suggest that most women currently do not appear to make informed decisions, with many women and their partners not being aware of the abovementioned implications of screening [4-7]. Ideally, women and couples should be accompanied and supported through the complex prenatal screening pathway. In practice, this is often difficult to achieve as not all individuals have a high level of health literacy, the time constraint for patient-clinician communication, especially in publicly funded health care setting, and concern of possible conflicts between recommendations from clinical best practice guidelines and couple’s preferences [8]. One way to overcome some of these difficulties is to use decision aids (DAs).

DAs, either passive or interactive, are advocated and used to assist shared decision-making. They provide unbiased and nondirective evidence regarding the available options, including the risks and benefits, and a means for individuals to determine and clarify their personal values that are relevant to outcomes [9,10]. Interactive digital decision aids (IDDAs), unlike passive DAs such as educational booklets, pamphlets, and web pages that provide static information, rely on user engagement by using 2-way communication, thereby allowing users to focus on specific aspects and thus should be an effective learning tool according to the cognitive learning theory [11-15].

Previous systematic reviews indicate that DAs used in pregnancy in general have an impact on informed decision-making by increasing knowledge and decreasing decisional conflict [9,16,17]. These studies mostly assessed the effectiveness of passive DAs, but with technological advances in recent years, we have seen an increased number of studies on IDDAs, and we have decided that more specific, updated evidence is needed in this one particular context [9,16,17]. By restricting the scope, the objective of this study was to assess the effect of IDDAs when used as an adjunct to standard care on pregnant women’s decision-making regarding prenatal screening.

## Methods

The PRISMA (Preferred Reporting Items for Systematic Reviews and Meta-Analyses) guidelines were followed to conduct and report this systematic review [18].

### Search Strategy and Selection of Studies

**Figure 1:**
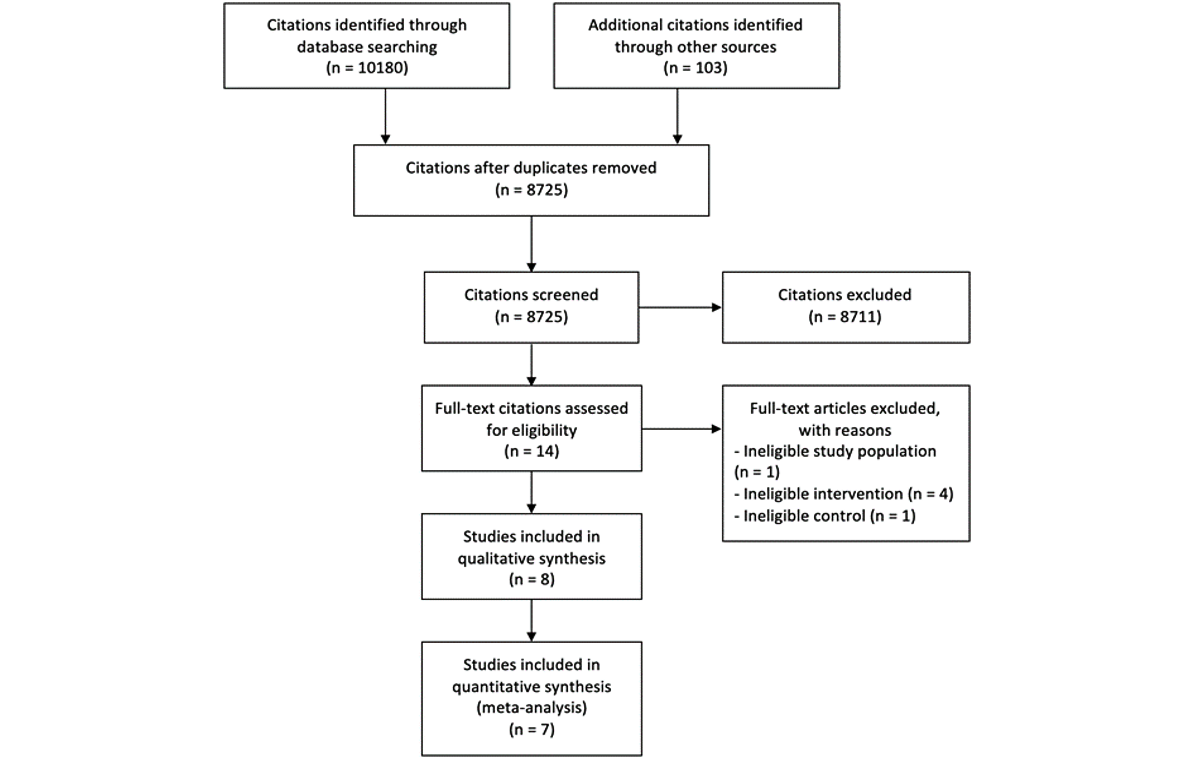
PRISMA (Preferred Reporting Items for Systematic Reviews and Meta-Analyses) flowchart of study selection process in the systematic review.

Four databases were searched for randomized controlled trials (RCTs), namely Cochrane Central Register of Controlled Trials (latest issue) in the Cochrane Library, MEDLINE Ovid (1946 to present), Embase Ovid (1910 to present), and PsycINFO Ovid (1806 to present) from inception until August 2021. Search strategies used in these databases can be found in Multimedia Appendix 1. We also searched the World Health Organization International Clinical Trials Registry Platform [19], the internet using Google Scholar, and the reference lists of all included studies for potentially eligible RCTs. Citations were retrieved from the aforementioned databases and other resources, exported into EndNote X9. After removing duplicates, titles and abstracts were first screened by a reviewer based on the inclusion and exclusion criteria. Then full texts were assessed to find eligible studies. It was then sent to a second reviewer for discussion. The screening and selection process follows the PRISMA flow diagram, as shown in Figure 1.

### Eligibility Criteria

RCTs, available as full-text studies, conference abstracts, and unpublished data, were included. Publication status and language were not part of the criteria.

Studies involving pregnant women who were making decisions regarding prenatal screening options for themselves were included. Studies with participants involved in proxy or passive decision-making or participants making hypothetical choices were excluded.

Studies with interventions being IDDAs, as an adjunct to standard care, were included. To be recognized as an IDDA, it had to fulfill the basic criteria suggested by the International Patient Decision Aid Standards (IPDAS) Collaboration and be both interactive and digital [11-14]. Studies failing to fulfill these criteria were excluded.

Studies with standard care as the control were included. Standard care mainly includes face-to-face consultation, counseling, provision of general information, and placebo intervention, but could vary from country to country. Studies focused on comparing different types of DAs were excluded.

Primary outcomes to establish the efficacy of IDDAs in terms of the quality of the decision-making process and the quality of the decision itself were (1) knowledge, assessed using specific questions concerning prenatal screening and (2) decisional conflict, measured with decisional conflict scale. Secondary outcomes included (1) accuracy of risk perceptions, (2) compatibility between final choice and personal values, and (3) how involved the patient is in decision-making assessed by Degner Control Preferences Scale [20].

### Data Extraction and Management

A reviewer extracted and entered the relevant data into Review Manager 5.4 (RevMan 5.4). Data extraction sheets were sent to a second reviewer for checking. Any ambiguities were also discussed with the second reviewer.

### Assessment of Risk of Bias

The risk of bias in included studies was assessed by a reviewer using the Cochrane risk-of-bias tool, according to the following domains: random sequence generation, allocation concealment, blinding of participants and personnel, blinding of outcome assessment, incomplete outcome data, selective outcome reporting, and any other bias [21]. Each potential source of bias was judged as conferring high, low, or unclear risk of bias [21]. Missing data and loss to follow-up were also assessed as one of the criteria of risk of bias. The initial assessment was checked by a second reviewer, a senior researcher from the team, who did not have any disagreement with the first reviewer.

### Strategy for Data Synthesis

Dichotomous data were analyzed based on the numbers of events and people in intervention and control groups. Data were entered into RevMan 5.4 to generate risk ratios (RRs) and 95% CIs. Continuous data were analyzed based on means, SDs, and number of people in intervention and control groups. Data were also entered into RevMan 5.4 to calculate standardized mean differences (SMDs) and 95% CIs. In case of nonavailability of SD, other methods to calculate SD, such as *t* value and Cohen *d*, were used. If outcomes were measured more than once over time, measurements at time points that were the closest to one another across studies were selected to be entered for analyses for better comparability.

Meta-analysis was conducted. A fixed-effects model was used to combine the included studies when there was no significant heterogeneity (*P* value of the Cochrane Q test>0.1, *I*^2^<30%) [22]. When there was significant, moderate or severe heterogeneity among included studies (*P* value of the Cochrane Q test<0.1, *I*^2^>30%), a random-effects model was used to estimate overall prevalence by incorporating the heterogeneity into calculation [22]. Subgroup analysis was used to detect the source of heterogeneity. Sensitivity analysis was also conducted in order to assess the effect of excluding studies that are of lower methodological quality. The analysis excluded studies with a “high risk of bias” for any one of the categories in the Cochrane risk-of-bias tool from meta-analysis.

PRISMA guidelines were followed when the systematic review was conducted and reported (see PRISMA checklist in Multimedia Appendix 2).

## Results

### Literature Search and Study Characteristics

Out of the 10,283 citations identified, 8 met the eligibility criteria and were included in this review (Figure 1). The 8 RCTs presented results from 4 different countries (5 studies from the United States, 1 study from the Netherlands, 1 study from Denmark, and 1 study from Hong Kong) and randomized 2981 individual participants. Of the 8 studies, 7 were included in our meta-analysis (see Multimedia Appendix 3). Leung et al’s [23] study was excluded from our meta-analysis because the only outcome in this study that matches with this review, “understanding” (considered equivalent to “knowledge”), was measured dichotomously and could not be quantitatively combined with the other 7 studies.

Six studies had on-site interventions (participants using IDDAs at the health care settings) [23-28] while 2 studies had off-site interventions (participants accessing the designed website were sent links to use for administering IDDAs remotely) [29,30].

Four studies used computers for administration [23,26-28]. Two studies sent websites’ links to participants for remote administration, and the devices used were therefore unknown [29,30]. One study had IDDA provided through a tablet-based application [25], and one study used IDDA but did not specify the method or platform for administration [24].

### Risk-of-Bias Assessment

All included studies had a low risk of bias regarding random sequence generation, allocation concealment, and blinding of outcome assessment. Blinding of outcome assessment was not done or mentioned in most of the included studies, but because the outcomes measured were mostly objective and did not involve much subjective interpretation by outcome assessors, it was decided that there were low risks of bias in this regard.

Most of the included studies did not mention blinding of participants and personnel and therefore had an unclear risk of bias. Most of them also had a low risk of attrition bias, but for 2 studies the risk of bias is unclear [24,26]. One study had a high level of no-response (>20%) and therefore had a high risk of bias in terms of incomplete outcome data [29]. Three of the studies were not registered with trial registries and did not have protocols available for assessment; they had an unclear risk of reporting bias. Some had relatively low participation rates, but the impact was unclear, explaining the classifications of “unclear” in “other bias”; only one study was classified as “high” in this regard [29] (Multimedia Appendix 4).

### Effect of Interventions

#### Knowledge

Seven studies assessed the effects of IDDAs on knowledge (eg, prenatal testing in general, specifically Down syndrome, or both). Different scales or modifications were used in each study, for example, Maternal Serum Screening Knowledge Questionnaire, Prenatal Screening Knowledge Survey, and Multidimensional Measure of Informed Choice. A random-effects model was used. The results showed that the use of IDDAs leads to a statistically significant improvement in pregnant women’s knowledge on prenatal screening, compared to standard care (SMD 0.58, 95% CI 0.26-0.90, *P*<.001). Although the performance of IDDAs was significantly better than standard care in terms of improving knowledge, there was a significant high level of heterogeneity (*I*^2^=94%, *P*<.001), which was further explored with subgroup analysis.

Leung et al [23], reporting a dichotomous outcome of “understanding,” which could be considered synonymous to “knowledge” in this context, found that 54.1% and 77.0% of participants reported they had no more questions on prenatal screening for Down syndrome in the control group (leaflet and video) and the intervention group (leaflet, video, and DA), respectively.

#### Decisional Conflict

Four studies assessed the effects of IDDAs on decisional conflict. All 4 studies used the decisional conflict scale as the outcome measurement, but each of them used different subscales or adaptations, thus SMDs were also used for this outcome. A fixed-effects model was used. Use of IDDAs demonstrated a statistically significant reduction in decisional conflict (SMD –0.15, 95% CI –0.25 to –0.05, *P*=.003). There was an insignificant low level of heterogeneity (*I*^2^=13%, *P*=.33).

#### Accuracy of Risk Perceptions

Two studies assessed the effects of IDDAs on the accuracy of risk perceptions in the following 2 aspects.

Number and percentage of correct estimate of procedure-related miscarriage risk: there was a statistically significant effect demonstrated using IDDAs (RR 1.19, 95% CI 1.04-1.36, *P*=.01). There was an insignificant moderate level of heterogeneity (*I*^2^=43%, *P*=.18).

Number and percentage of correct estimate of Down syndrome risk: no significant effect was found for the use of IDDAs (RR 1.84, 95% CI 0.85-3.97, *P*=.12). There was also a significant high level of heterogeneity (*I*^2^=94%, *P*<.001).

A random-effects model was used in both aspects. However, subgroup analyses would not be practical for this outcome because only 2 studies were included.

#### Compatibility Between Final Choice and Personal Values

Only one study measured whether the participants’ final choice of screening was consistent with their personal values [30]. In the intervention group, 94.2% made consistent choices, whereas 92.7% made consistent choices in the comparison group, which had rather similar levels. The effect demonstrated in the study was not significant (*P*=.64).

#### How Involved the Patient is in Decision-making

The outcome “how involved the patient is in decision-making” was not found in any of the included studies and thus would not be described or analyzed.

**Figure 2:**
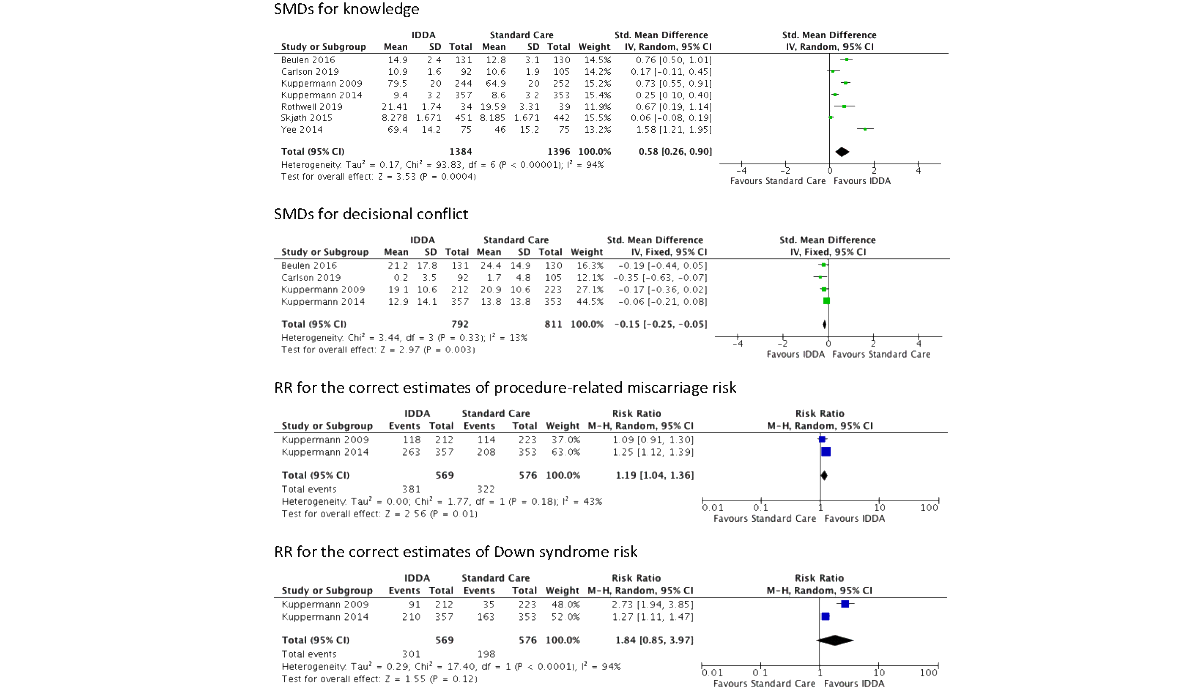
Forest plots for the outcomes knowledge, decisional conflict, the correct estimates of procedure-related miscarriage risk, and the correct estimates of Down syndrome risk. IDDA: interactive digital decision aid.

The forest plots for the outcomes of knowledge, decisional conflict, the correct estimate of procedure-related miscarriage risk, and the correct estimate of Down syndrome risk are presented in Figure 2.

### Subgroup Analysis

We performed subgroup analysis for the outcome “knowledge,” according to whether the intervention was conducted on site or off site, whether the control adopted standard care, and whether the outcome was measured immediately after the treatment. Only the last subgroup analysis showed difference between groups.

**Figure 3:**
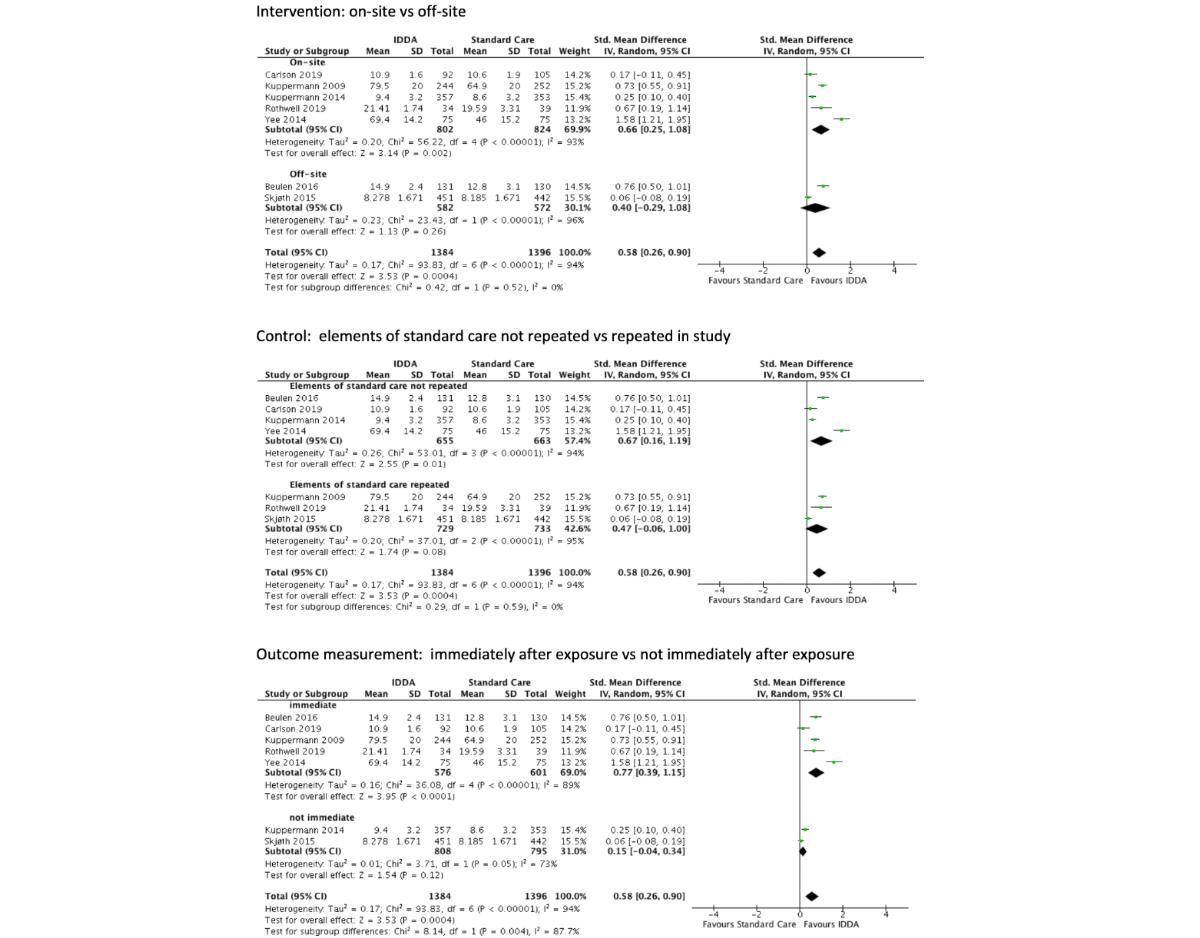
Subgroup analysis for knowledge. IDDA: interactive digital decision aid.

An immediate assessment, as opposed to later, generally showed a higher level of knowledge (SMD 0.77, 95% CI 0.39-1.15 vs SMD 0.15, 95% CI 0.04 to 0.34). This could be interpreted that knowledge level under this context of prenatal screening decision-making would reduce overtime. Additionally, significant high level of heterogeneity still remains in each subgroup (*I*^2^=89%, *P*<.001 and *I*^2^=73%, *P*=.05), meaning that the heterogeneity was still not completely resolved and there might be other sources that were not identified. The forest plots of subgroup analysis are presented in Figure 3.

### Sensitivity Analysis

Skjøth et al’s [29] study was removed from sensitivity analysis because high risks of bias were found in the following two criteria: “incomplete outcome data” and “other bias”. Results of sensitivity analysis showed that low-quality study did not affect the outcomes too much (original pooled SMD 0.58, 95% CI 0.26-0.90, *P*<.001 vs sensitivity analysis pooled SMD 0.68, 95% CI 0.33-1.02, *P*<.001). The forest plot for sensitivity analysis can be seen in Multimedia Appendix 5.

## Discussion

### Principal Findings

In this review, we performed a systematic literature search and meta-analysis to evaluate the effectiveness of IDDAs on pregnant women’s decision-making regarding prenatal screening. We found that, when compared to standard care alone, the use of IDDAs increased knowledge, decreased decisional conflict, and increased accuracy of procedure-related miscarriage risk perceptions. Subgroup analyses found that the timing of outcome measurement had an impact on knowledge, with later measurements resulting in lower knowledge levels. However, no conclusive results could be drawn on the accuracy of risk perceptions on Down syndrome, coherence between final choice and personal values, and patient involvement.

### Strengths and Limitations

This is the first systematic review, to our knowledge, that is focused on this specific type of DAs and IDDAs, in the context of prenatal screening decision-making. We included RCTs that used IDDAs fulfilling the basic criteria set out by the IPDAS Collaboration [11]. To evaluate the impact of IDDAs on the quality of decision-making process and the quality of the decision itself, we looked into a number of primary and secondary outcomes as suggested by the IPDAS Collaboration [20].

However, our findings have certain limitations. First, the heterogeneity of the outcome knowledge was substantial (*I*^2^=94%) and could not be resolved through subgroup analysis. Although the evidence for IDDAs leading to an improvement in knowledge was fairly strong, the extent of the effect could not be completely determined due to the high heterogeneity shown. It is also worth noticing that for the subgroup analyses conducted for knowledge, because of the small number of studies in each subgroup, conclusions on subgroup effects may not be entirely reliable either, and careful interpretation of the data is required.

Second, the pooled SMD of decisional conflict was relatively small (SMD –0.15), although the 95% CI did not intersect with the line of no effect (95% CI –0.25 to –0.05), the effect of reducing decisional conflict was relatively weak and not as meaningful.

Third, the results of meta-analyses conducted for the 2 aspects of accuracy of risk perceptions were based on only 2 studies. Although this was not much of an issue per se, these 2 studies were conducted by the same first author affiliated with the same institution and in the same state (California) in the United States, meaning that the generalizability of the results of meta-analysis conducted in this review on this outcome would be relatively low [27,28]. Moreover, the fact that there are only 2 studies in the meta-analysis of this outcome makes it difficult to investigate the potential reasons of heterogeneity. The small number of studies makes the *I*^2^ value more prone to bias and may not be meaningful [31]. There is also clinical heterogeneity, such as differences in educational attainment and ethnicity, and methodological diversity, such as differences in the designs of these 2 studies, both of which could contribute to the statistical heterogeneity *I*^2^ manifested, but it is difficult to pinpoint.

Moreover, apart from the insignificant effect of IDDAs on estimating Down syndrome risk, the RR for correct estimates of procedure-related miscarriage risk was quite low (RR 1.19, 95% CI 1.04-1.36), similar to the low SMD for decisional conflict, caution is needed when interpreting and concluding an increase in accuracy.

Some other limitations of this review are as follows: the Grading of Recommendations, Assessment, Development, and Evaluations framework should ideally be performed in addition to the risk-of-bias assessment in order to make sure the quality of the review can be further strengthened, and only a small number of studies is being included, and therefore a funnel plot could not be done to explore potential publication bias. Although we did not set up language as part of the inclusion criteria, only English studies were identified, and only Western populations were included in our meta-analyses, which could lead to problems with generalizability.

### Implications

IDDAs can be effectively used as an adjunct to routine care in facilitating pregnant women’s informed decision-making regarding prenatal screening. The use of digital technology in DAs offers an opportunity to incorporate a range of flexible applications such as texts, animations, images, audios, videos, games, social networking tools, and risk calculators to provide health information [32]. These digital DAs facilitate the information delivery at a time and place preferred by women. Furthermore, the incorporation of interactive elements in patient-centered digital DAs can complement counseling from health care professionals and provide decision support.

However, results of the meta-analyses of DAs to support decision-making were highly heterogeneous [9,13,17]. Even with the restriction of the DAs’ scope to IDDAs in the setting of prenatal screening, we still did not manage to resolve this issue. One of the potential sources, although not possible to be explored in detail in this review, is the variations between the contents of different DAs: such variations include not only the platforms, mode of administration, or certain features such as interactivity, but more importantly, the qualities of the DAs themselves and the materials they incorporate. With these potential variations, each DA may have effects of widely different extents on the outcomes measured, which can be an explanation of such heterogeneities.

In fact, several objective criteria have been developed for the assessment of the qualities of DAs, for instance, the IPDAS instrument [17] by the IPDAS Collaboration; yet, they are still not widely adopted [13]. All included studies in this review had their own DAs, only one study stated that the DA they developed was according to the IPDAS instrument, which actually did not provide much detail on the DA or the score the DA got when assessed with the IPDAS instrument [30]. Another study merely acknowledged the need for values clarification exercise, citing the IPDAS Collaboration, whereas all other included studies did not mention any criteria or standards that their DAs were based upon [24]. Such insufficient utilization of assessment criteria could also be identified by the fact that the outcomes such as user involvement, despite being suggested by the IPDAS Collaboration, were very seldom assessed in primary studies on DAs, and none of the included studies in this review assessed this outcome at all. This underreporting of user involvement in publications by the DAs’ developers has also been identified by the IPDAS Collaboration [33]. The problems of limited reporting of DAs’ content, development process, delivery, and effectiveness evaluation measures have also been mentioned in previous studies [34-36].

We suggest that such variations in the qualities and contents of DAs, without objective assessments, were one of the major sources of heterogeneities that could not be discovered in this review. Such practices should be improved, with a wider use of aforementioned assessment instruments. However, this does not mean a complete standardization of DAs, as it might hinder originality and creativity as well as lower specificities of DAs on each type of medical decision on specific conditions. Besides, it is important to ensure that the effectiveness standards of the IPDAS Collaboration are met, with the measures of decision quality and decision process criteria [37]. Such comparison against an existing benchmark would be helpful in developing a more standardized approach to measurement. On the other hand, further investigation would be needed to gain further insight as to whether this is indeed a major reason leading to such heterogeneities.

### Conclusions

IDDAs have the potential to be a convenient and effective adjunct to the current standards of pregnancy care, especially for women’s decision-making in prenatal screening. Although evidence for benefits such as an increase in knowledge, decrease in decisional conflict, and increase in accuracy of certain aspects of risk perceptions were established, more research is still needed. Future developments of DAs, including IDDAs, should use objective quality assessment instruments such as the IPDAS instrument more frequently. This could facilitate evaluation of their effectiveness by measuring against existing benchmarks, which could thereby help improve the qualities of evidence in this regard in the future. Future studies or reviews could also put more effort into identifying the reasons of heterogeneities in the use of DAs, not just IDDAs, particularly whether qualities and contents of DAs constitute a major source. Lastly, studies could also look into ways to integrate the use of IDDAs into different clinical conditions of prenatal care around the world, preparing for its potential future use in obstetric care.

## Appendix

Multimedia Appendix 1 Search strategies used in the four electronic databases.

Multimedia Appendix 2 Preferred Reporting Items for Systematic reviews and Meta-Analyses (PRISMA) checklist.

Multimedia Appendix 3 Characteristics of included studies.

Multimedia Appendix 4 Risk of bias summaries.

Multimedia Appendix 5 Sensitivity analysis.

## Abbreviations

DA: decision aid
IDDA: interactive digital decision aid
IPDAS: International Patient Decision Aid Standards
RCT: randomized controlled trial
RevMan 5.4: Review Manager 5.4
RR: risk ratios
SMD: standardized mean differences
PRISMA: Preferred Reporting Items for Systematic Reviews and Meta-Analyses

## Data Availability

The data that support the findings of this study are available on request from the corresponding author. The data are not publicly available due to privacy or ethical restrictions.
