# Supplementary material for: Effectiveness of Interactive Digital Decision Aids in Prenatal Screening Decision-making: Systematic Review and Meta-analysis
Source: J Med Internet Res. 2023 Mar 14;25:e37953. doi: 10.2196/37953 (PMC10131906; doi:10.2196/37953)
Supplement: Multimedia Appendix 1 [file jmir_v25i1e37953_app1.docx]

**Multimedia Appendix 1: Search strategies used in the four electronic databases**

**CENTRAL via the Cochrane Library**

#1 decision support techniques:kw

#2 decision support systems clinical:kw

#3 decision making:kw

#4 choice behavior:kw

#5 ((decision* or decid*) near/3 (aid* or support* or tool* or technolog* or system* or program* or algorithm* or technique* or instrument* or intervention* or material* or guide* or counsel*)):ti,ab,kw

#6 shared decision making:ti,ab,kw

#7 ((inform*) next (choice* or decision* or consent*)):ti,ab,kw

#8 directive counsel*:ti,ab,kw

#9 #1 or #2 or #3 or #4 or #5 or #6 or #7 or #8

#10 computer:ti,ab,kw

#11 interactive:ti,ab,kw

#12 ((interact* or computer* or phone* or digital or electronic) near/2 (decision aid* or decision making or communication* or tool* or program* or game* or software* or guide*)):ti,ab,kw

#13 (internet* or online or digital or electronic or webcast* or web-cast*):ti,ab,kw

#14 ((cell* or mobile*) next (phone* or device*)):ti,ab,kw

#15 (smartphone* or smart-phone* or mobile* or tablet*):ti,ab,kw

#16 (android or windows or linux or ios or mac or ipad* or ipod* or iphone* or i-pad* or i-pod* or i-phone*):ti,ab,kw

#17 ((mobile* or phone*) next (app*)):ti,ab,kw

#18 (multimedi* or multi-medi*):ti,ab,kw

#19 #10 or #11 or #12 or #13 or #14 or #15 or #16 or #17 or #18

#20 ((prenatal or antenatal or pre-natal or ante-natal) near/3 (screen* or test* or diagnos* or detect*)):ti,ab,kw

#21 prenatal:ti,ab,kw

#22 pregnant:ti,ab,kw

#23 maternal:ti,ab,kw

#24 (fetal or fetus* or foetal or foetus* or feti or foeti or embryo*):ti,ab,kw

#25 (aneuploid* or trisom* or chromosom* or down* syndrome*):ti,ab,kw

#26 (terminat* or deliver* or labour* or labor* or birth* or born or obstetrics or gynaecology or gynecology or obgyn or ob-gyn):ti,ab,kw

#27 #20 or #21 or #22 #23 or #24 or #25 or #26

#28 randomi?ed:ti,ab,kw

#29 placebo*:ti,ab,kw

#30 randomly:ti,ab,kw

#31 trial:ti,ab,kw

#32 group*:ti,ab,kw

#33 clinical:ti,ab,kw

#34 control*:ti,ab,kw

#35 #28 or #29 or #30 or #31 or #32 or #33 or #34

#36 #9 and #19 and #27 and #35 in Trials

**MEDLINE Ovid**

| 1 | decision support techniques/ |
| --- | --- |
| 2 | decision support systems clinical/ |
| 3 | decision making/ |
| 4 | choice behavior/ |
| 5 | ((decision* or decid*) adj3 (aid* or support* or tool* or technolog* or system* or program* or algorithm* or technique* or instrument* or intervention* or material* or guide* or counsel*)).tw. |
| 6 | shared decision making.tw. |
| 7 | informed choice*.tw. |
| 8 | informed decision*.tw. |
| 9 | informed consent*.tw. |
| 10 | directive counseling.tw. |
| 11 | or/1-10 |
| 12 | computer.tw. |
| 13 | interactive.tw. |
| 14 | ((interact* or computer* or phone* or digital or electronic) adj2 (decision aid* or decision making or communication* or tool* or program* or game* or software* or guide*)).tw. |
| 15 | (internet* or online or digital or electronic or webcast* or web-cast*).tw. |
| 16 | ((cell* or mobile*) adj1 (phone* or device*)).tw. |
| 17 | (smartphone* or smart-phone*).tw. |
| 18 | mobile.tw. |
| 19 | tablet.tw. |
| 20 | (android or windows or linux or ios or mac or ipad* or ipod* or iphone* or i-pad* or i-pod* or i-phone*).tw. |
| 21 | ((mobile* or phone*) adj1 app*).tw. |
| 22 | (multimedi* or multi-medi*).tw. |
| 23 | or/12-22 |
| 24 | ((prenatal or antenatal or pre-natal or ante-natal) adj3 (screen* or test* or diagnos* or detect*)).tw. |
| 25 | prenatal.tw. |
| 26 | pregnan*.tw. |
| 27 | wom#n.tw. |
| 28 | female*.tw. |
| 29 | maternal.tw. |
| 30 | (fetal or fetus* or foetal or foetus* or feti or foeti or embryo*).tw. |
| 31 | (aneuploid* or trisom* or chromosom* or down* syndrome*).tw. |
| 32 | (terminat* or deliver* or labour* or labor* or birth* or born or obstetrics or gynaecology or gynecology or obgyn or ob-gyn).tw. |
| 33 | or/24-32 |
| 34 | randomized controlled trial.pt. |
| 35 | controlled clinical trial.pt. |
| 36 | randomized.ab. |
| 37 | placebo.ab. |
| 38 | randomly.ab. |
| 39 | trial.ti. |
| 40 | groups.ab. |
| 41 | or/34-40 |
| 42 | exp animals/ not humans.sh. |
| 43 | 41 not 42 |
| 44 | 11 and 23 and 33 and 43 |

**Embase Ovid**

| 1 | decision support system/ |
| --- | --- |
| 2 | decision aid/ |
| 3 | decision making/ |
| 4 | choice behavior/ |
| 5 | ((decision* or decid*) adj3 (aid* or support* or tool* or technolog* or system* or program* or algorithm* or technique* or instrument* or intervention* or material* or guide* or counsel*)).tw. |
| 6 | shared decision making.tw. |
| 7 | informed choice*.tw. |
| 8 | informed decision*.tw. |
| 9 | informed consent*.tw. |
| 10 | directive counseling.tw. |
| 11 | or/1-10 |
| 12 | computer.tw. |
| 13 | interactive.tw. |
| 14 | ((interact* or computer* or phone* or digital or electronic) adj2 (decision aid* or decision making or communication* or tool* or program* or game* or software* or guide*)).tw. |
| 15 | (internet* or online or digital or electronic or webcast* or web-cast*).tw. |
| 16 | ((cell* or mobile*) adj1 (phone* or device*)).tw. |
| 17 | (smartphone* or smart-phone*).tw. |
| 18 | mobile.tw. |
| 19 | tablet.tw. |
| 20 | (android or windows or linux or ios or mac or ipad* or ipod* or iphone* or i-pad* or i-pod* or i-phone*).tw. |
| 21 | ((mobile* or phone*) adj1 app*).tw. |
| 22 | (multimedi* or multi-medi*).tw. |
| 23 | or/12-22 |
| 24 | ((prenatal or antenatal or pre-natal or ante-natal) adj3 (screen* or test* or diagnos* or detect*)).tw. |
| 25 | prenatal.tw. |
| 26 | pregnan*.tw. |
| 27 | wom#n.tw. |
| 28 | female*.tw. |
| 29 | maternal.tw. |
| 30 | (fetal or fetus* or foetal or foetus* or feti or foeti or embryo*).tw. |
| 31 | (aneuploid* or trisom* or chromosom* or down* syndrome*).tw. |
| 32 | (terminat* or deliver* or labour* or labor* or birth* or born or obstetrics or gynaecology or gynecology or obgyn or ob-gyn).tw. |
| 33 | or/24-32 |
| 34 | crossover-procedure/ |
| 35 | double-blind procedure/ |
| 36 | randomized controlled trial/ |
| 37 | single-blind procedure/ |
| 38 | (random* or factorial* or crossover* or cross over* or placebo* or (doubl* adj blind*) or (singl* adj blind*) or assign* or allocat* or volunteer*).tw. |
| 39 | or/34-38 |
| 40 | 11 and 23 and 33 and 39 |

**PsycINFO Ovid**

| 1 | decision support system/ |
| --- | --- |
| 2 | decision making/ |
| 3 | choice behavior/ |
| 4 | ((decision* or decid*) adj3 (aid* or support* or tool* or technolog* or system* or program* or algorithm* or technique* or instrument* or intervention* or material* or guide* or counsel*)).tw. |
| 5 | shared decision making.tw. |
| 6 | informed choice*.tw. |
| 7 | informed decision*.tw. |
| 8 | informed consent*.tw. |
| 9 | directive counseling.tw. |
| 10 | or/1-9 |
| 11 | computer.tw. |
| 12 | interactive.tw. |
| 13 | ((interact* or computer* or phone* or digital or electronic) adj2 (decision aid* or decision making or communication* or tool* or program* or game* or software* or guide*)).tw. |
| 14 | (internet* or online or digital or electronic or webcast* or web-cast*).tw. |
| 15 | ((cell* or mobile*) adj1 (phone* or device*)).tw. |
| 16 | (smartphone* or smart-phone*).tw. |
| 17 | mobile.tw. |
| 18 | tablet.tw. |
| 19 | (android or windows or linux or ios or mac or ipad* or ipod* or iphone* or i-pad* or i-pod* or i-phone*).tw. |
| 20 | ((mobile* or phone*) adj1 app*).tw. |
| 21 | (multimedi* or multi-medi*).tw. |
| 22 | or/11-21 |
| 23 | ((prenatal or antenatal or pre-natal or ante-natal) adj3 (screen* or test* or diagnos* or detect*)).tw. |
| 24 | prenatal.tw. |
| 25 | pregnan*.tw. |
| 26 | wom#n.tw. |
| 27 | female*.tw. |
| 28 | maternal.tw. |
| 29 | (fetal or fetus* or foetal or foetus* or feti or foeti or embryo*).tw. |
| 30 | (aneuploid* or trisom* or chromosom* or down* syndrome*).tw. |
| 31 | (terminat* or deliver* or labour* or labor* or birth* or born or obstetrics or gynaecology or gynecology or obgyn or ob-gyn).tw. |
| 32 | or/23-31 |
| 33 | control:.tw. |
| 34 | random:.tw. |
| 35 | exp treatment/ |
| 36 | or/33-35 |
| 37 | 10 and 22 and 32 and 36 |
