# Supplementary material for: Effectiveness of Interactive Digital Decision Aids in Prenatal Screening Decision-making: Systematic Review and Meta-analysis
Source: J Med Internet Res. 2023 Mar 14;25:e37953. doi: 10.2196/37953 (PMC10131906; doi:10.2196/37953)
Supplement: Multimedia Appendix 3 [file jmir_v25i1e37953_app3.docx]

**Multimedia Appendix 3: Characteristics of included studies**

| **Beulen 2016** | | |
| --- | --- | --- |
| Methods | Randomized controlled trial  Web-based multimedia decision aid (and standard prenatal care) vs standard prenatal care only | |
| Participants | The study location was the Netherlands  157 participants were randomized to the intervention group and 157 participants were randomized to the control group | |
| Interventions | Intervention: Web-based multimedia decision aid  Intervention group participants received a personal link providing access to a web-based multimedia decision aid on prenatal testing in addition to usual care. This decision aid was developed according to the International Patient Decision Aid Standards (IPDAS). A value clarification exercise was also  Control: Usual care  Control group participants received standard prenatal care including delivery of information on prenatal screening for chromosomal abnormalities in early pregnancy through standardized brochure and counselling provided by a certified obstetric healthcare provider | |
| Outcomes | Primary outcome: informed decision-making regarding prenatal testing (modified scale of Multidimensional Measure of Informed Choice)  Secondary outcomes: knowledge, attitudes, prenatal test utilisation, value-consistency, decisional conflict (Decisional Conflict Scale), decisional regret, and anxiety (scale of the Spielberger State Trait Anxiety Inventory)  Outcomes were measured  Pregnant women randomized to the intervention group were sent a personal link providing access to the web-based multimedia decision aid for the duration of 1 week. One week after randomization, during the process of decision-making, participants were invited to complete the first of two web-based questionnaires (Q1). Thus, participants randomized to the intervention group did not have the decision aid available while completing this questionnaire. The invitation to complete a follow-up questionnaire was sent at around 28 weeks of gestation, when the decisional process was completed and, where applicable, prenatal testing for chromosomal abnormalities was performed and the outcome was known (Q2).  Q1 was done before intervention after randomization and Q2 was done at 28weeks of gestation. | |
| Notes | - | |
| **Risk of bias** | | |
| **Bias** | **Authors’ judgement** | **Support/evidence for judgement** |
| Random sequence generation | Low risk | ~~"After obtaining informed consent, participants were allocated to the control or intervention group by~~ A computer-generated randomisation ~~(p1410, Procedures)~~ |
| Allocation concealment | Low risk | “computer-generated randomisation” (p1410, Procedures) |
| Blinding of participants and personnel | Unclear risk | Not mentioned in the study |
| Blinding of outcome assessment | Low risk | Not mentioned about blinding of the outcome assessment in the study but outcomes are objectively measured and not subject to interpretation |
| Incomplete outcome data | Low risk | No data that was available was omitted. Maximum missing data was less than 10% (<20%), difference of missing data between groups was small |
| Selective outcome reporting | Unclear risk | Protocol not available, no indication that it was registered in trials registry |
| Other bias | Low risk | No other sources of bias identified |
| **Carlson 2019** | | |
| Methods | Randomized controlled non-inferiority trial | |
| Participants | The study location was USA  92 participants were randomized to the intervention group and 105 participants were randomized to the control group | |
| Interventions | Intervention: Novel computerized decision aid  Intervention group participants received self-administered the decision aid within the app before the schedule of seeing a genetic counselor for a discussion of aneuploidy screening and testing.  Control: Usual care  Control group participants received counselling of aneuploidy screening and testing with genetic counselors after discussion with their prenatal care providers | |
| Outcomes | Primary outcome: knowledge score (modified Maternal Serum Screening Knowledge Questionnaire with updated screening modalities)  Secondary outcomes: decisional conflict (validated low literacy Decisional Conflict Scale) following decision aid completion, decisional conflict following decision aid completion and genetic counseling, change in knowledge score after genetic counseling in women who used the decision aid, choice of testing, and pursuit of invasive testing with high risk results.  Knowledge assessment (baseline, questionnaire 1)) was performed immediately following the randomization. Control group participants completed the knowledge and decisional conflict assessments (questionnaire 2) after genetic counselling. Intervention group participants completed the knowledge and decisional conflict assessments after using the self-administered the decision aid within the app (questionnaire 2) and after genetic counselling (questionnaire 3) | |
| Notes |  | |
| **Risk of bias** | | |
| **Bias** | **Authors’ judgement** | **Support/evidence for judgement** |
| Random sequence generation | Low risk | The authors state "Via a coin-flip algorithm within the app, women were randomly assigned to group 1 (control group) or group 2 (decision aid group)" (p924, Materials and Methods) |
| Allocation concealment | Low risk | The authors state"Investigators were not involved in developing the randomization scheme within the app" (p924, Materials and Methods) |
| Blinding of participants and personnel | Unclear risk | The authors state "Blinding was not performed as it was not pragmatic for this study; many women were eager to discuss the decision aid with the genetic counselor when it was used" (p924, Materials and Methods) and “Genetic counselors were educated prior to study initiation that their counseling should not be modified based on group assignment”  But it is unsure whether this would have any impact on the study |
| Blinding of outcome assessment | Low risk | Not mentioned about blinding of the outcome assessment in the study but outcomes are objectively measured and not subject to interpretation |
| Incomplete outcome data | Low risk | There was no dropouts from the study in both the control group and intervention group |
| Selective outcome reporting | Low risk | The trial was registered  in ClinicalTrials**.**gov (NCT02991729) and all relevant results appear to be reported |
| Other bias | Unclear risk | The participation rate was 54% (197/365) and its impact on the study is unclear |
| **Kuppermann 2009** | | |
| Methods | Randomized controlled trial | |
| Participants | The study location was USA  244 participants were randomized to the intervention group and 252 participants were randomized to the control group | |
| Interventions | Intervention: Prenatal Testing Decision-Assisting Tool (PT)  Intervention group participants received Prenatal Testing Decision-Assisting Tool with values clarification exercises  Control: Standard educational booklet | |
| Outcomes | Primary outcomes: knowledge of prenatal testing and the conditions for which it tests, awareness of procedure-related miscarriage and age-adjusted Down syndrome risks, intervention satisfaction, and decisional conflict  Secondary outcomes: decision regret, intervention effect, and satisfaction with decision-making involvement (Among women aged >=35 years, use of invasive testing was also included as a secondary outcome)  Outcomes were measured at baseline, immediately after viewing the intervention, 1-2 weeks later through telephone, and at 26-30 gestational weeks, with no specific reasons of measuring which outcomes at which timepoint(s) | |
| Notes | - | |
| **Risk of bias** | | |
| **Bias** | **Authors’ judgement** | **Support/evidence for judgement** |
| Random sequence generation | Low risk | The authors state "the interviewer opened an opaque envelope containing the randomization assignment and started the computer program to which the participant had been assigned (PT Tool or the control intervention). Randomization was stratified by age (younger than 35 years or 35 years or older) and language (English or Spanish). " but do not specify how the random sequence was generated |
| Allocation concealment | Low risk | The authors state "the interviewer opened an opaque envelope containing the randomization assignment" (p55, Materials and Methods |
| Blinding of participants and personnel | Unclear risk | No mention of blinding |
| Blinding of outcome assessment | Low risk | Not mentioned about blinding of the outcome assessment in the study but outcomes are objectively measured and not subject to interpretation |
| Incomplete outcome data | Low risk | Similar attrition, not too substantial (<20% for both groups) |
| Selective outcome reporting | Unclear risk | The trial was registered  in ClinicalTrials**.**gov (NCT00686062) but outcome information was not provided in the registered study records and therefore could not be compared with the published study |
| Other bias | Unclear risk | The participation rate was 54% (588/1080) and its impact on the study is unclear |
| **Kuppermann 2014** | | |
| Methods | Randomized controlled trial | |
| Participants | The study location was California  375 participants were randomized to the intervention group and 369 participants were randomized to the control group | |
| Interventions | Intervention: A computerized, interactive decision-support guide  Intervention group participants were provided access to Prenatal Testing: Exploring Your Options located at the interview site, a decision-support guide which was formatted as an audio-, video-, and text-based interactive computer program including values clarification exercises  Control: Usual care  Control group participants received usual care per current guidelines | |
| Outcomes | Primary outcome: use of invasive prenatal diagnostic testing  Secondary outcomes: testing strategy undergone, knowledge, risk comprehension, decisional conflict, and decision regret  Outcomes such as use of invasive prenatal diagnostic testing were assessed by reviewing participants’ medical records, other outcomes such as knowledge were measured during the follow-up telephone interview at 24-36 gestational weeks | |
| Notes | This study does not only look into the effect of DA (enhanced information and values clarification), but also removal of financial barriers | |
| **Risk of bias** | | |
| **Bias** | **Authors’ judgement** | **Support/evidence for judgement** |
| Random sequence generation | Low risk | "A computer-generated random allocation sequence |
| Allocation concealment | Low risk | The authors state "The randomization code was not available to any study-related personnel until data analysis was complete" (p1211, Methods: Procedures) |
| Blinding of participants and personnel | Unclear risk | Blinding of personnel was achieved, but blinding of participants was not mentioned. Due to the nature of the intervention, it was not possible to blind the participants to randomization status. Its impact on the study is unclear |
| Blinding of outcome assessment | Low risk | Not mentioned about blinding of the outcome assessment in the study but outcomes are objectively measured and not subject to interpretation |
| Incomplete outcome data | Low risk | Used modified intention-to-treat analysis; similar attrition in both groups |
| Selective outcome reporting | Low risk | Registered with ClinicalTrials.gov (NCT00505596), outcomes seem to be the same as the registered records |
| Other bias | Unclear risk | The trial was registered  in ClinicalTrials**.**gov (NCT00505596), and all relevant outcomes appear to be reported |
| **Leung 2004** | | |
| Methods | Randomized controlled trial | |
| Participants | The study location was Hong Kong  100 participants were randomized to the intervention group and 101 participants were randomized to the control group | |
| Interventions | Intervention: An interactive multimedia decision aid (IMDA)  Intervention group participants received information on prenatal screening for Down syndrome through an information leaflet, video commentary and the IMDA in one closed room  Control: Video and information leaflet  Control group participants received information on prenatal screening for Down syndrome through an information leaflet and video commentary | |
| Outcomes | Primary outcome: final uptake of the screening test (integrated or serum screening) for Down syndrome  Secondary outcomes: initial decision (made after viewing the video with or without browsing the decision aid), understanding, and satisfaction with the information that they had received  Primary outcome was collected from the medical record after the choice was made by the participant. Secondary outcomes were collected through questionnaires after viewing the video from both groups and after browsing the decision aid from intervention group | |
| Notes | - | |
| **Risk of bias** | | |
| **Bias** | **Authors’ judgement** | **Support/evidence for judgement** |
| Random sequence generation | Low risk | Sequence generation was not specified. Eligible patients were randomized on a 1 : 1 |
| Allocation concealment | Low risk | Sealed, opaque envelopes were used |
| Blinding of participants and personnel | Unclear risk | Given the nature of the interventions, participants were not blind to their allocation |
| Blinding of outcome assessment | Low risk | Not mentioned about blinding of the outcome assessment in the study but outcomes are objectively measured and not subject to interpretation |
| Incomplete outcome data | Low risk | Number of people who dropped out are accounted for 6/100 (6%) of intervention group and 3/101 (3%) of control group. Overall 4% dropout which is small |
| Selective outcome reporting | Unclear risk | No protocol provided; trial not registered |
| Other bias | Low risk | No other sources of bias identified |
| **Rothwell 2019** | | |
| Methods | Randomized controlled trial | |
| Participants | The study location was USA  40 participants were randomized to the intervention group and 39 participants were randomized to the control group | |
| Interventions | Intervention: A game-based decision aid  Intervention group participants were given the brochure-based decision and a game-based decision aid (“The Meaning of Screening”)  Control: Standard of care  Control group participants were given the brochure-based decision aid during their first obstetric visit in the clinic as part of routine care along with any physician communication, as per the practice of the individual clinician | |
| Outcomes | Primary outcome: knowledge  Secondary outcomes: values and screening behaviour  Outcomes were measured with survey immediately after interventions | |
| Notes | - | |
| **Risk of bias** | | |
| **Bias** | **Authors’ judgement** | **Support/evidence for judgement** |
| Random sequence generation | Low risk | A computer generated simple randomization sequence |
| Allocation concealment | Low risk | "computer-generated simple randomization sequence" (p323, Materials and Methods: Procedures) |
| Blinding of participants and personnel | Unclear risk | Given the nature of the interventions, participants were not blind to their allocation |
| Blinding of outcome assessment | Low risk | Not mentioned about blinding of the outcome assessment in the study but outcomes are objectively measured and not subject to interpretation |
| Incomplete outcome data | Unclear risk | Number of people who dropped out are accounted for 6/40 (15%) of intervention group and 0/39 (0%) of control group which was explained by technical issues. Overall 8% dropout which is small |
| Selective outcome reporting | Low risk | The trial was registered  in ClinicalTrials**.**gov (NCT03441672), and all relevant outcomes appear to be reported |
| Other bias | Unclear risk | The enrolment rate was 54% (80/149) and its impact on the study is unclear |
| **Skjøth 2015** | | |
| Methods | Randomized controlled trial | |
| Participants | The study location was Denmark  577 participants were randomized to the intervention group and 578 participants were randomized to the control group | |
| Interventions | Intervention: An interactive website  Intervention group participants were offered standard information and a specially designed eHealth tool (an interactive website) containing information about Down syndrome screening  Control: Standard information  Control group participants were offered standard information in the form of a hospital booklet and access to the hospital website about Down syndrome screening | |
| Outcomes | Primary outcome: an informed choice about Down syndrome screening (Multidimensional Measure of Informed Choice)  Secondary outcomes: knowledge and attitude  Outcomes were measured with questionnaire that was sent to participants 6 weeks after nuchal translucency test (approximately 18 gestational weeks), but varies across participants sometimes because of late responses | |
| Notes | - | |
| **Risk of bias** | | |
| **Bias** | **Authors’ judgement** | **Support/evidence for judgement** |
| Random sequence generation | Low risk | A computer-generated randomization with a block size of 10 |
| Allocation concealment | Low risk | The authors state “During the study, the block size was blinded to the recruiting midwife and to the researchers.” |
| Blinding of participants and personnel | Low risk | The authors state” To maintain comparability and blinding as best as possible: (i) women in the control group were invited to use the general website of the hospital; (ii) neither women in the intervention nor the control group were aware of the constitution of the other group; (iii) staff in the Maternal Fetal Medicine Clinic were blinded to randomization; and (iv) researchers were not part of the recruitment or randomization process.” |
| Blinding of outcome assessment | Low risk | The authors state "Only in assessing outcomes were the researchers aware of the composition of the two groups" |
| Incomplete outcome data | High risk | Used intention-to-treat analysis. In control group, the response rate to the questionnaire is 79% (459/578) and reporting rate of not using the intervention is 96% (442/459). In intervention group, the response rate to the questionnaire is 78% (451/577) and reporting rate of using the intervention is only 29% (132/451). |
| Selective outcome reporting | Low risk | The trial was registered  in ClinicalTrials**.**gov (NCT01889550), and all relevant outcomes appear to be reported |
| Other bias | High risk | Numbers of "reporting having used the intervention" or "not" contradict with the actual uptake of intervention/control, huge numbers of the contradicting ones were not included (e.g. approximately 3 quarters of those in intervention group were left out for analysis) |
| **Yee 2014** | | |
| Methods | Randomized controlled trial | |
| Participants | The study location was USA  75 participants were randomized to the intervention group and 75 participants were randomized to the control group | |
| Interventions | Intervention: An interactive computerized information aid  Intervention group participants received a self-guided interactive computerized patient education tool in a private clinic room in addition to standard of care counseling for prenatal screening and diagnosis  Control: Standard care provider-based counseling  Control group participants received standard care with provider-based counseling | |
| Outcomes | Outcome: knowledge  Outcome was measured immediately after interventions, and also 2-4 weeks after randomization | |
| Notes | Values clarification exercise was not explicitly mentioned in the study but it could be conducted given the highly interactive features of the computer programme (the intervention) | |
| **Risk of bias** | | |
| **Bias** | **Authors’ judgement** | **Support/evidence for judgement** |
| Random sequence generation | Low risk | A computer-generated random number table |
| Allocation concealment | Low risk | "computer-generated random number table" (p553, Methods) |
| Blinding of participants and personnel | Unclear risk | Given the nature of the interventions, participants were not blind to their allocation |
| Blinding of outcome assessment | Low risk | The authors state "Open-ended questions were judged to be correct by a single investigator (WAG) who was unaware of a participants’ group assignment" (p553, Methods) and the outcomes are objectively measured and not subject to interpretation |
| Incomplete outcome data | Unclear risk | Number of people who lost to follow-up are accounted for 16/75 (21%) intervention and 11/75 (15%) control and they are similar for both arms. Overall 18% dropout. Reasons for not completing were not presented. We therefore rate this study as being at unclear risk of bias |
| Selective outcome reporting | Low risk | The trial was registered  in ClinicalTrials**.**gov (NCT01176019), and all relevant outcomes appear to be reported |
| Other bias | Unclear risk | The participation rate was 57% (150/261) and its impact on the study is unclear |
