# Supplementary material for: Effectiveness of Interactive Digital Decision Aids in Prenatal Screening Decision-making: Systematic Review and Meta-analysis
Source: J Med Internet Res. 2023 Mar 14;25:e37953. doi: 10.2196/37953 (PMC10131906; doi:10.2196/37953)

**Multimedia Appendix 4: Risk of bias summaries**

Fig A4-1: Risk of bias summary presented as percentages across all included studies


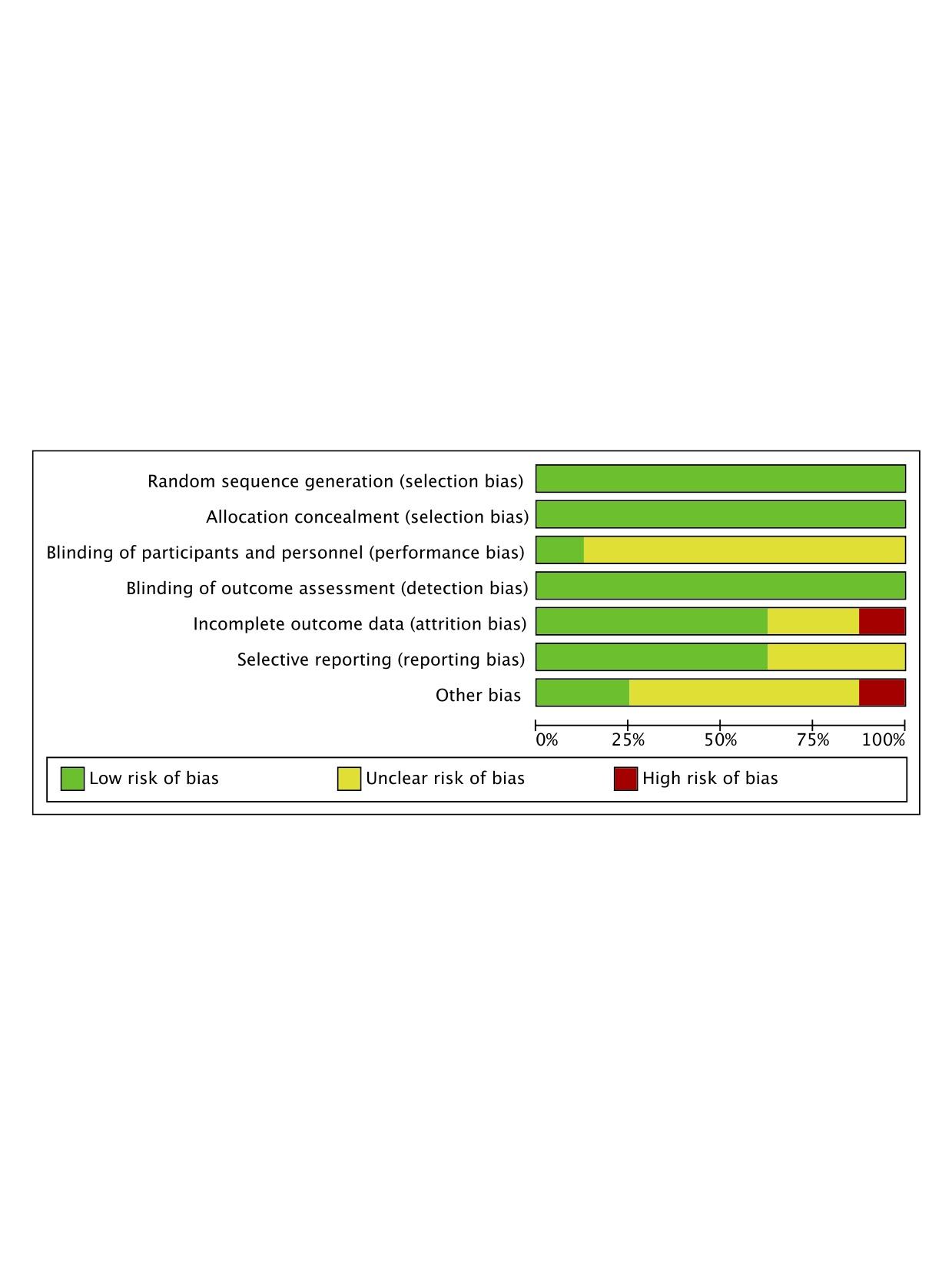


Fig A4-2: Risk of bias summary for each included study


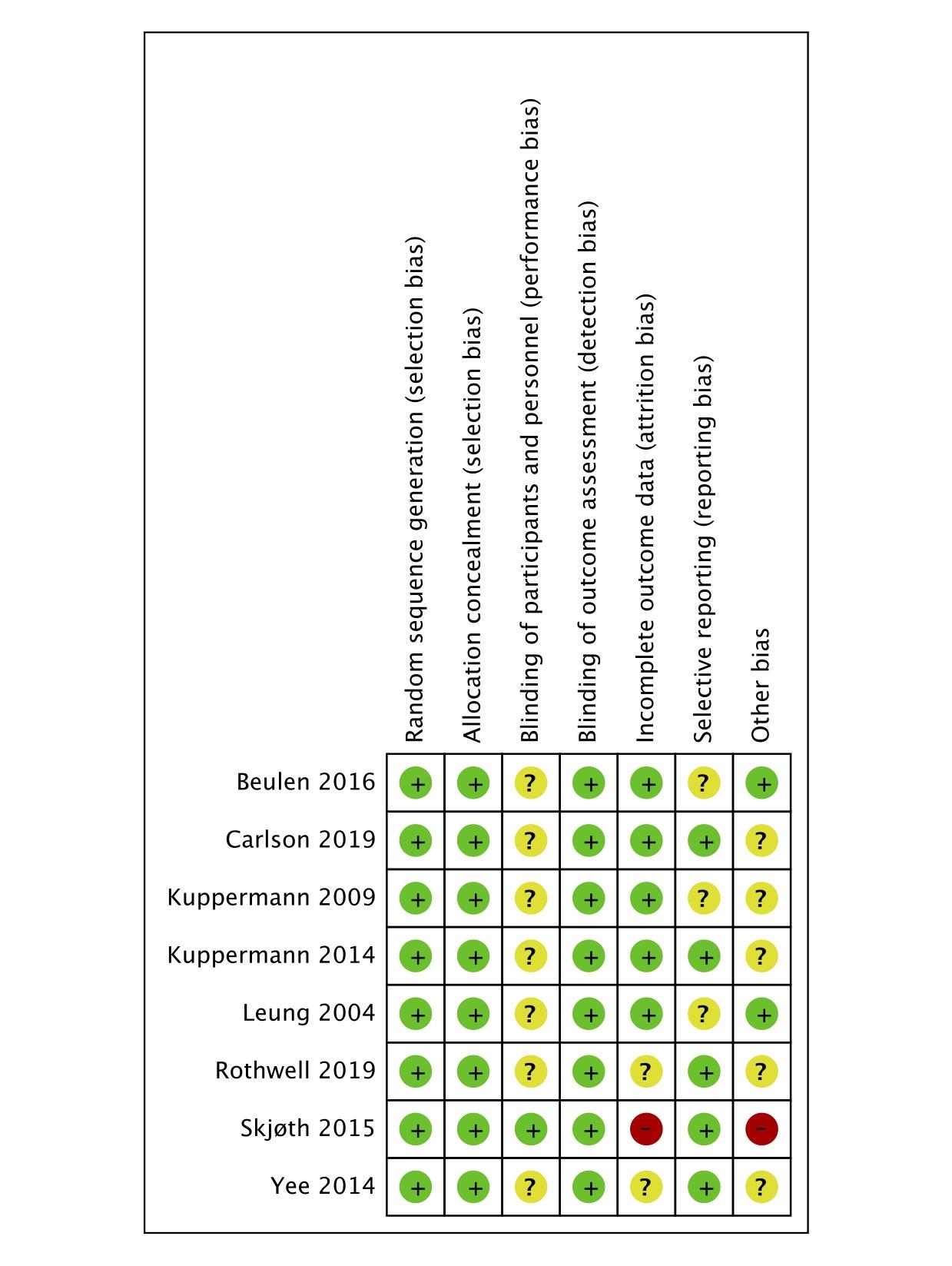

Supplement: Multimedia Appendix 4 [file jmir_v25i1e37953_app4.docx]
