# Supplementary material for: Effectiveness of Interactive Digital Decision Aids in Prenatal Screening Decision-making: Systematic Review and Meta-analysis
Source: J Med Internet Res. 2023 Mar 14;25:e37953. doi: 10.2196/37953 (PMC10131906; doi:10.2196/37953)

**Multimedia Appendix 5: Sensitivity analysis (Skjøth 2015 removed)**

**Fig A5-1: Sensitivity analysis (Skjøth 2015 removed)**


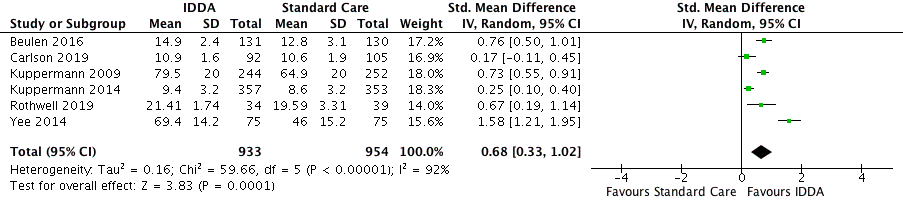

Supplement: Multimedia Appendix 5 [file jmir_v25i1e37953_app5.docx]
